# The effects of nutritional support team intervention on postoperative immune function, nutritional statuses, inflammatory responses, clinical outcomes of elderly patients with gastric cancer

**DOI:** 10.1186/s12893-022-01784-9

**Published:** 2022-10-03

**Authors:** Jiansheng Chen, Liling Zou, Wenxing Sun, Junfeng Zhou, Qingliang He

**Affiliations:** 1 Department of Gastrointestinal Surgery, The First Affiliated Hospital of Fujian Medical University, No.20 Chazhong Road, Fuzhou, 350005 Fujian People’s Republic of China; 2 The First Clinical Medical College, Fujian Medical University, Fuzhou, 350122 Fujian People’s Republic of China

**Keywords:** Nutrition support team, Gastric cancer, Elderly patients, Immune function, Long-term prognosis

## Abstract

**Background:**

To explore the effects of nutrition support team (NST) intervention on elderly patients with gastric cancer (GC).

**Methods:**

The elderly GC patients (tumor stage I/II/III), admitted to our department from January 2015 to September 2021, were retrospectively analyzed and divided into NST group and traditional nutrition (TN) group according to nutritional management methods. The immune, inflammatory, nutrition-related indices, postoperative recovery and long-term prognosis of two groups were analyzed.

**Results:**

A total of 258 elderly GC patients were included (NST group, n = 125; TN group, n = 133). After propensity score matching (PSM) in ratio of 1:1, 73 pairs of patients were matched. There were statistically significant differences in CD3 and CD4 level postoperative one month and IgG level postoperative one week between NST group and TN group (P < 0.05). There was no significant differences in serum CRP and IL-6 levels preoperative one day, postoperative one week and one month between two groups (P > 0.05). There were significant differences in body mass index (BMI) between the two groups postoperative one month (P < 0.05). The rate of infectious complications in TN group was significantly higher than that in NST group (P < 0.05). There was no statistically significant differences in 3-year relapse-free survival (RFS) or 3-year overall survival (OS) between NST group and TN group (P > 0.05).

**Conclusions:**

Compared with TN management, NST intervention might be benefit to the immune function recovery and nutritional status, but there was no evidence that NST could improve the prognosis of elderly GC patients.

## Background

Gastric cancer (GC) is the fifth most common cancer and the third most common cause of cancer death in the world, with more than 1 million new cases every year [1]. The incidence of GC has obvious regional differences, 40% of which are concentrated in China [2, 3]. Age is one of the highest risk factors for cancer, and the majority of solid tumors are regarded age-related diseases. According to the investigation of scholars, the incidence of GC increases from the age of 50, and the main age of onset is between 60 and 70 years old, indicating that the elderly constitute the main body of GC patients [4, 5]. Cancer management in the elderly can be complex; comorbidity, multidrug and age-related physiological changes can affect the perioperative management and subsequent treatment choices of cancer [6, 7]. However, most current studies on gastric cancer treatment usually aimed at adults over 18 years old, and some do not include gastric cancer patients over 80 years old, let alone a specific population of elderly gastric cancer patients [8].

Different from younger GC patients, elderly GC patients are often accompanied by consumption and chronic diseases, such as diabetes, hypertension and chronic obstructive pulmonary disease [9]. In addition, the high metabolism of tumor makes malnutrition become very common in elderly GC patients. Severe malnutrition will further affect the body’s immunity and important organ functions, and also increase the incidence of complications after GC surgery [10]. The nutritional status is even closely related to the long-term prognosis of GC patients [11, 12]. Therefore, high attention should be paid to the nutritional status of elderly GC patients before and after surgery. Currently, there is limited information on nutrition support team (NST) intervention for elderly GC patients. Thus, this study aimed to explore the effect of NST intervention mode on postoperative immune function and long-term prognosis of elderly GC patients.

## Materials and methods

### Patients

The elderly GC patients (tumor stage I/II/III) who underwent laparoscopic radical gastrectomy in Department of Gastrointestinal Surgery of the First Affiliated Hospital of Fujian Medical University from January 2017 to September 2021 were retrospectively analyzed. According to the ways of nutritional management, the patients were divided into NST group and traditional nutrition group (TN group). Inclusion criteria of patients were: (1) Age ≥ 60 years old (The legal age for senior citizens in China is 60 years or older.); (2) Before operation, gastroscopy confirmed that the pathology was GC, and CT showed no distant organ metastasis, so laparoscopic radical gastrectomy was performed; (3) No neoadjuvant therapy was received before operation; (4) No severe organ dysfunction and other intolerable diseases. Exclusion criteria were: (1) Patients with a history of gastric surgery; (2) American Society of Anesthesiologists (ASA) IV–V patients; (3) Combined with mental disorders and other diseases that could not be treated; (4) Patients with incomplete information. This study was approved by the Branch for Medical Research and Clinical Technology Application, Ethics Committee of the First Affiliated Hospital of Fujian Medical University (MTCA, ECFAH of FMU [2015] No.084). The patients and their families signed the informed consent forms after learning about the experimental processes.

### Nutritional management

#### Nutritional support team (NST) mode

*Composition and management mode* The NST consists of 1 clinician, 1 nurse, 1 clinical pharmacist and 1 nutritionist. The nutritional support mode was that the nutritionist, clinical pharmacist and nurse followed the ward round of the surgical treatment group every day. After checking the patients, the nutritionist and clinical pharmacist given specific nutrition support plans for each patient, and the nurses carried out necessary nutrition support education for the patients and implement the nutrition support plans.

*Assessment of nutritional status* Clinicians and nutritionists conducted nutritional risk screening for patients within 24 h after admission. Nutritional status of subjects was assessed using the scored Nutrition Risk Screening (NRS) 2002 [13] and Patient-Generated Subjective Global Assessment (PG-SGA) tool [14]. NRS 2002 was an objective nutritional risk screening tool that includes four assessment aspects (anthropometric measurements, recent weight change, dietary intake and disease severity), scores range from 0 to 7, with values equal to or greater than 3 indicating nutritional risk. PG-SGA is an effective tool for the assessment of tumor patient-specific nutritional status, includes two specific content: patient self-assessment and medical staff assessment, score of 0–1 means no malnutrition and no intervention required; when score is ≥ 2 points, it indicate that there is malnutrition and different nutritional interventions need to be carried out according to the severity of malnutrition. The patient’s medical history, examination results, anthropometry and other results were collected to construct a complete nutritional medical record. The key nutritional indicators included height, weight and body mass index (BMI). According to patients’ nutritional status at admission, patients received different lengths of time of nutritional support therapy, nutrition prescriptions was issued by dietitians and jointly reviewed by clinicians and pharmacists. Nurses were responsible for recording the discomfort and complications of patients. After comprehensive evaluation of postoperative condition, test indicators and gastrointestinal recovery, nutritional requirements and nutritional forms of the patients were jointly evaluated by nutritionists and clinicians.

*Nutritional intervention strategy* Nutrition support enteral nutrition (EN) was preferred, combined with parenteral nutrition (PN) or total parenteral nutrition (TPN) when EN was intolerant, the condition did not permit, or the EN could not meet energy and fluid volume and electrolyte requirements. Since the energy consumption of each elderly patient could not be accurately measured, we set 20–30 kcal/(kg·d) as the target amount according to the guideline for energy consumption of elderly inpatients [15–17]. The proportion of the three nutrients was 50–60% carbohydrate, 20–30% fat and 15–20% protein. As for the selection of oral nutritional supplements (ONS), enteral nutrition powder, enteral nutrition suspension and enteral nutritional emulsion were selected according to whether patients had diabetes and tolerance to the products. In addition, if the patient’s condition permitted and the family members were able to make their own diet, quantified individual diet could be formulated according to the individual situation of each patient.

### Traditional nutritional management

Clinicians used NRS2002 and PG-SGA tools to screen the nutritional risk of hospitalized patients, determined the indications of nutritional support treatment and selected nutritional preparations needed for patients based on own experience. They needed to deal with the complications related to the patient’s nutritional support and adjust the nutritional support program alone, and invited nutritionists and clinical pharmacists to consult for treatment assistance if necessary.

### Data collection

On admission, the baseline data were collected. The following main outcome indicators were collected one day before surgery, one week and one month after surgery: immune-related indicators (serum levels of CD3, CD4, CD8, CD4/CD8, IgA and IgG) and stress inflammatory factors (serum C-reactive protein (CRP) and interleukin-6 (IL-6) levels). The secondary outcome measures were nutritional indicators (BMI) and postoperative situation (the first flatus time and defecation time after surgery, time to liquid diet, complications, postoperative hospital stay, Clavien-Dindo complication grading system [18], hospitalization cost). The prognostic indicators, 3-year relapse-free survival (RFS), 3-year overall survival (OS) were also analyzed.

### Definition of infectious complications

Postoperative infectious complications mainly include: intra-abdominal infection, incision infection, anastomotic leakage, postoperative pneumonia, catheter-related blood stream infection. The diagnostic criteria for infectious complications were: (1) Intra-abdominal infection refers to patients with abdominal symptoms, imaging examinations suggest intra-abdominal infection or abdominal puncture to extract pus, and the result of bacterial culture in ascites is positive; (2) Incision infection refers to the appearance of redness, swelling, heat, pain, and bloody or purulent exudate in the surgical incision; (3) Anastomotic leakage is defined as an upper gastrointestinal angiography showing contrast medium spillage from the gastrointestinal tract and the drainage of digestive juices, purulent fluid, or gas through a drainage tube; (4) Postoperative pneumonia was defined as new or progressive infiltrates, consolidations, or ground-glass opacities on lung X-ray or CT, With respiratory symptoms or positive sputum culture; (5) Catheter-related blood stream infection refers to an infection caused by an intravascular catheter that occurs within 48 h of the patient's application of a central venous catheter, and there is a laboratory report confirming a bloodstream infection or clinical sepsis.

### Statistical analysis

SPSS 25.0 package (SPSS Inc., Armonk, NY, United States) was used to perform the analysis. PSM module was used to perform propensity score matching (PSM) according to 1:1. The normal distribution of the measurement data was expressed by means ± standard deviation (SD). The independent T-test was used for comparison of normal distribution measurement data, and non-parametric test was used for comparison of non-normal distribution measurement data. The Chi-square test or Fisher’s exact test were used to compare the categorical variables between the two groups. The RFS and OS were compared between the two groups by Kaplan–Meier and log-rank tests. P < 0.05 was considered statistically significant.

## Results

### General information

A total of 258 elderly tumor stage I/II/III GC patients were included (NST group, n = 125; TN group, n = 133). There were statistically significant differences in history of diabetes, surgical site and tumor stage between two groups (all P < 0.05, Table 1). After PSM, 73 pairs of patients were finally matched, and there was no statistically significant difference in age, gender, BMI, history of diabetes and hypertension, NRS2002/PG-SGA score, nutritional status, and ASA grade between 2 groups (all P > 0.05), suggesting that there was no significant difference in body status between the two groups. There was no statistical difference in surgical site, methods of reconstruction of digestive tract, scope of lymph node dissection and tumor stage (P > 0.05), further indicating that there was no significant difference in disease status between two groups and it was comparability (Table 2).

**Table 1:** Comparison of the general patient data between two groups

Basic data	TN group (n = 133)	NST group (n = 125)	P-value
Age (year, n)			
60–69	79	88	0.17
70–79	47	31	
80–89	7	6	
Gender (Male/Female, n)	89/44	95/30	0.11
BMI (kg/m^2^)	21.8 ± 3.0	21.9 ± 3.0	0.49
Diabetes (n)			
Yes	29	15	0.04
No	104	110	
Hypertension (n)			
Yes	40	26	0.09
No	93	99	
Nutritional status (n)			
Normal	106	87	0.06
Malnourished	27	38	
ASA grade (n)			
I	93	82	0.46
≥ II	40	43	
Surgical site (n)			
Proximal	16	22	0.04
Distal	62	39	
Full stomach	55	64	
Scope of lymph node dissection (n)			
D1	9	12	0.41
D2	124	113	
Tract reconstruction (n)			
Billroth II	18	27	0.10
Roux-en-Y	115	98	
Tumor stage (n)			
I	38	48	< 0.01
II	62	33	
III	33	44	

*TN* traditional nutrition, *NST* nutrition support team, *BMI* body mass index, *NRS2002* nutritional risk screening, *PG-SGA* patient-generated subjective global assessment, *ASA* American Society of Anesthesiologists

**Table 2:** Comparison of the general patients’ data between two groups after propensity score matching (PSM)

Variable	TN group (n = 73)	NST group (n = 73)	P-value
Age (year, n)			
60–69	51	55	0.60
70–79	19	17	
80–89	3	1	
Gender (Male/Female, n)	54/19	59/14	0.27
BMI (kg/m^2^)	22.1 ± 2.7	22.1 ± 2.8	0.60
Diabetes (n)			
Yes	14	7	0.17
No	59	66	
Hypertension (n)			
Yes	19	15	0.43
No	54	58	
Nutritional status (n)			
Normal	60	57	0.53
Malnourished	13	16	
ASA grade (n)			
I	45	49	0.49
≥ II	28	24	
Surgical site (n)			
Proximal	6	13	0.16
Distal	33	25	
Full stomach	34	35	
Scope of lymph node dissection (n)			
D1	3	4	0.70
D2	70	69	
Tract reconstruction (n)			
Billroth II	12	10	0.64
Roux-en-Y	61	63	
Tumor stage (n)			
I	25	27	0.76
II	26	28	
III	22	18	

*TN* traditional nutrition, *NST* nutrition support team, *BMI* body mass index, *NRS2002* nutritional risk screening, *PG-SGA* patient-generated subjective global assessment, *ASA* American Society of Anesthesiologists

### Compliance with nutritional support in two groups

Compliance in this study refers to patients’ compliance with medical advice and guidance related to nutritional support, which includes the type of EN and PN taken, the amount of energy and fluid required, and compliance with the schedule. In the NST group, one patient stopped PN infusion due to fever caused by PN infusion, two patients had reduced PN infusion due to progressive liver transaminase elevation, Two patients were intolerant to EN. In TN group, two patients had reduced PN due to central venous catheter infection and inability to tolerate PN, one patient had skin rash due to PN, and one patient delayed early postoperative feeding due to diarrhea caused by EN.

### Changes in the related immune indices in the two groups

There were statistically significant differences in terms of the serum level of CD3 and CD4 postoperative one month and IgG level postoperative one week between NST group and TN group (all P < 0.05), while there was no significant difference in other indices. In the intra-group comparison, the levels of serum CD3, CD4, CD8, IgA and IgG in the TN group postoperative one week and the levels of serum CD4 and CD8 postoperative one month showed statistically significant differences compared with those before surgery (all P < 0.05). The levels of CD3, CD4, CD8, IgA and IgG in NST group postoperative one week were significantly different from those before operation (all P < 0.05), and the levels of serum CD3, CD4 and CD8 postoperative one month showed statistically significant differences compared with those before surgery in NST group (all P < 0.05). There was no significant difference in the above indices postoperative one month compared with those before surgery (Table 3).

**Table 3:** Comparison of immune indices and inflammatory factors between two groups

Variable	TN group (n = 73)			NST group (n = 73)		
	POD	POW	POM	POD	POW	POM
CD3 (/μL)	1876.0 ± 550.4	1429.6 ± 486.3^b^	1653.1 ± 465.7^a^	1883.2 ± 546.1	1433.9 ± 489.2^b^	1659.6 ± 468.7^ab^
CD4 (/μL)	919.3 ± 289.6	752.2 ± 230.4^b^	781.8 ± 249.0^ab^	922.2 ± 291.4	750.1 ± 230.9^b^	783.7 ± 249.7^ab^
CD8 (/μL)	750.9 ± 273.7	622.1 ± 221.0^b^	660.4 ± 209.9^b^	755.1 ± 272.8	621.4 ± 222.6^b^	658.3 ± 209.6^b^
CD4/CD8	1.5 ± 0.8	1.4 ± 0.7	1.3 ± 0.6	1.5 ± 0.8	1.4 ± 0.8	1.3 ± 0.6
IgA (g/L)	2.7 ± 1.2	2.2 ± 0.9^b^	2.4 ± 0.9	2.6 ± 1.2	2.2 ± 0.9^b^	2.4 ± 0.9
IgG (g/L)	12.7 ± 1.2	9.2 ± 2.0^ab^	10.6 ± 2.2	12.7 ± 1.1	9.2 ± 2.1^ab^	10.5 ± 2.3
CRP (mg/L)	7.9 ± 3.9	17.3 ± 2.5^b^	6.5 ± 3.4	8.3 ± 3.7	17.2 ± 2.5^b^	6.7 ± 3.4^b^
IL-6 (pg/mL)	2.8 ± 1.5	4.0 ± 3.6	2.6 ± 1.3^b^	2.9 ± 1.5	3.6 ± 2.3^b^	2.5 ± 1.3

*POD* preoperative one day, *POW* postoperative one week, *POM* postoperative one month, *CRP* C-reactive protein, *IL-6* interleukin-6. ^a^Represents comparison between groups at the same time, P < 0.05; ^b^represents comparison between postoperative and preoperative within the same group, P < 0.05

### The inflammatory mediator levels and nutritional indices in two groups

In terms of stress inflammatory factors, there was no significant difference in serum CRP and IL-6 levels preoperative one day, postoperative one week and one month between two groups (P > 0.05). In the intra-group comparison, there were statistically significant differences in serum CRP level postoperative one week and serum IL-6 level postoperative one month in the TN group (P < 0.05), serum CRP and IL-6 level in NST group were statistically postoperative one week (P < 0.05), and serum CRP level postoperative one month was also statistically significant, all compared with preoperative in the same group (Table 3). In terms of nutritional indicators, there were significant differences in BMI between the two groups postoperative one month (P < 0.05). In the intra-group comparison, BMI in the TN group and NST group was no significantly decreased postoperative one week and one month compared with that before surgery respectively (P > 0.05, Table 4).

**Table 4:** Comparison of nutritional indicators between two groups

BMI (kg/m^2^)	TN group (n = 73)			NST group (n = 73)		
	POD	POW	POM	POD	POW	POM
< 18.5	12	17	16^a^	7	9	5^a^
18.5–22.9	31	36	30^a^	36	41	36^a^
≥ 23.0	30	20	27^a^	30	23	32^a^

*BMI* body mass index, *POD* preoperative one day, *POW* postoperative one week, *POM* postoperative one month

^a^Represents comparison between groups at the same time, P < 0.05

### Postoperative indicators and long-term prognosis of two groups

**Fig. 1:**
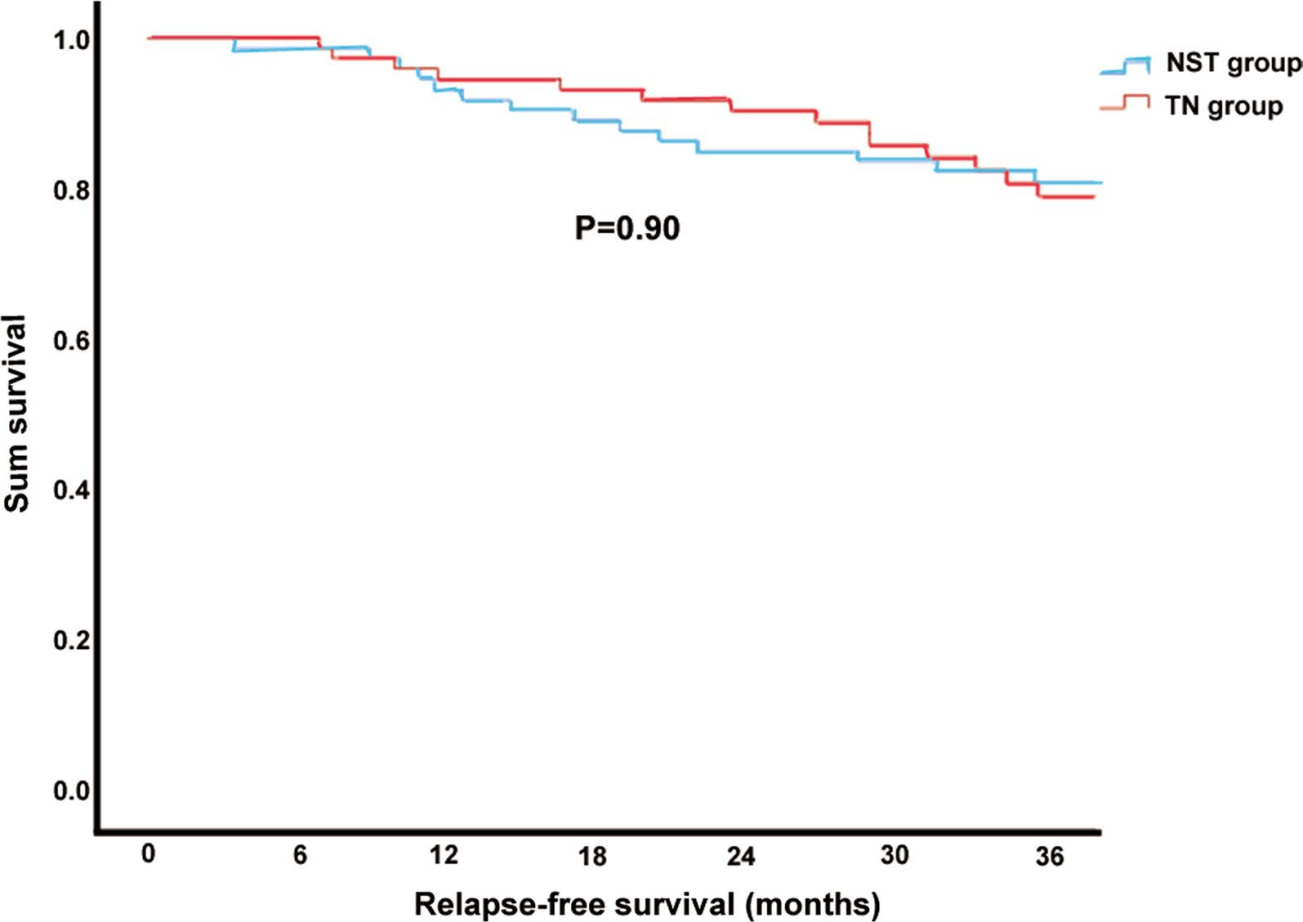
Three-year relapse-free survival of two group

**Fig. 2:**
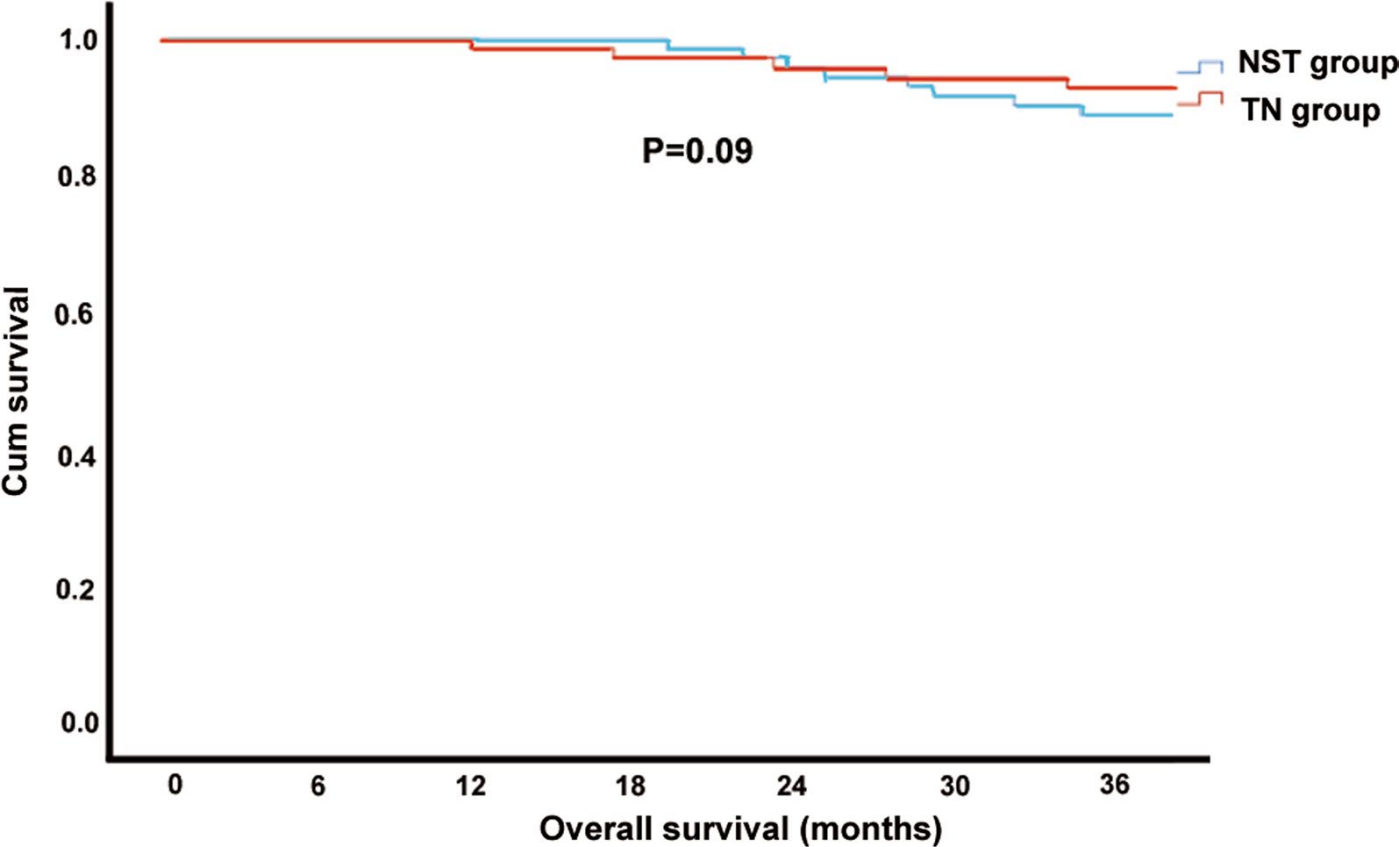
Three-year overall survival of two group

There were no significant differences in recovery time of the first flatus and defecation, time to liquid diet, C-D grade and hospitalization cost between the NST group and TN group (P > 0.05). Although there was no significant difference in overall postoperative complications between the two groups, further analysis of complications subdivided into infectious complications and non-infectious complications showed that the rate of infectious complications in the TN group was significantly higher than that in the NST group (P < 0.05, Table 5). Furthermore, **t**here was no statistically significant differences in 3-year RFS or 3-year OS between NST group and TN group (P > 0.05, Figs. 1, 2).

**Table 5:** Comparison of postoperative indicators between two groups

Variable	TN group (n = 73)	NST group (n = 73)	P-value
First flatus time (days)	2.1 ± 1.5	2.2 ± 1.5	0.47
First defecation time (days)	4.3 ± 1.7	4.3 ± 1.7	0.44
liquid diet time (days)	3.0 ± 1.6	3.0 ± 1.7	0.11
Complications (n)	10	6	0.29
Infectious complications	9	1	0.02
Non-infectious complications	1	5	0.21
C-D grade (n)			
I–II	8	5	0.70
III–IV	2	1	
Hospitalization cost (RMB)	69,722.4 ± 15,461.6	69,726.5 ± 15,540.6	0.62

C-D grade, Clavien-Dindo complication grading system

## Discussion

In TN management mode, there is no full-time physician responsible for the patient’s nutritional support. Clinicians usually evaluate the nutritional status and grasp the indications of the patient’s nutritional support alone. Due to the lack of systematic learning of nutrition theory, the selection of PN has become non-standard, and there is a lack of comprehensive understanding of the complications of the patient’s nutritional support. The NST originated from the successful application of parenteral nutrition by Dr. Dudrick’s team, who showed that PN infusion could be used to treat short bowel syndrome induced by beagle models, and was subsequently applied to humans [19]. The formula of PN is complex, and infusion often brings different degrees of complications. Therefore, the implementation, monitoring and adjustment of treatment plan cannot be separated from the close collaboration of multiple disciplines, thus the concept of NST came into being.

Through daily to weekly discussions, the team develops an individualized nutritional treatment plan for patients. Further, one study has reported NST intervention may affect the improvement of activities of daily living (ADL) in older patients undergoing in-patient rehabilitation [20]. We believe that the key to a team running smoothly and benefiting patients is communication, each member of the team has different responsibilities, and the focus and information obtained in their work are also different, through regular joint rounds and discussions, it is conducive to the mutual exchange of information and the improvement of team collaboration. In addition, nutrition education throughout the whole process of pre-operative, post-operative and post-discharge is also a content that cannot be ignored, through regular study, we pass on the knowledge we have learned to patients and their families, thereby so as to change their misunderstandings about postoperative diet and awareness of nutritional support.

Some studies have shown that the incidence of malnutrition in patients with malignant tumor can be as high as 40–80%, especially in GC and esophageal cancer [21–24]. The elderly GC patients have low organ reserve, the insufficient nutrients will affect the metabolism of immune cells, leading to the prominent of immune function decline. Liu et al. have reported that the postoperative early enteral nutrition program could reduce the incidence of postoperative complications and improve clinical outcomes [25]. In this study, the immunity related-indices CD3, CD4, IgA and IgG showed a general trend of decreasing after surgery and then gradually increasing. The last follow-up level of those indices in NST group was closer to the preoperative level. There were significant differences in postoperative levels of CD3 and CD4 between NST and TN groups. Although not all observed immune indexes were different, this also suggested that NST could help to improve the immune indexes of elderly gastric cancer patients in the short term after surgery. Notably, CD3, CD4, CD8 and immunoglobulin are commonly used to evaluate immune function [26].

In the analysis of postoperative recovery, the severity of postoperative complications in the TN group and NST group was not significantly different, although there was no significant difference in the overall complication rate between the two groups, the incidence of infectious complications in NST group was significantly lower than that in TN group, so we can think that the implementation of NST could reduce the occurrence of postoperative infectious complications under the condition that the hospitalization cost is roughly the same. At present, the research on nutritional support for GC patients mainly focuses on the short-term outcomes such as postoperative intestinal recovery and hospital stay [27, 28]. This study further explored the survival difference of patients receiving different nutritional support methods. It was found that there was no significant difference in the long-term prognosis between the two different nutritional management methods, which was inconsistent with the conclusions of previous studies that malnutrition was one of the independent risk factors for poor prognosis of elderly GC patients [29]. It may be related to the limited number of cases included in this study.

## Conclusion

This study confirmed that the NST intervention model could improve the immune function and reduce the incidence of infectious complications, which has important clinical significance for the nutritional management of elderly GC patients in the future. However, this study also has some shortcomings. Although the PSM analysis method was used in this study, the bias caused by unknown confounding factors could not be controlled. This study was a retrospective study with a small sample size, and the further clarification is still needed in a multi-center, large-sample prospective randomized controlled study.

## Data Availability

All data generated or analyzed were included in this published article.

## Abbreviations

NST: Nutrition support team
GC: Gastric cancer
TN: Traditional nutrition
PSM: Propensity score matching
OS: Overall survival
ASA: American Society of Anesthesiologists
NRS: Nutrition risk screening
PG-SGA: Patient-generated subjective global assessment
BMI: Body mass index
ALB: Albumin
EN: Enteral nutrition
PN: Parenteral nutrition
TPN: Total parenteral nutrition
ONS: Oral nutritional supplements
CRP: C-reactive protein
IL-6: Interleukin-6
C–D: Clavien–Dindo
RFS: Relapse-free survival
OS: Overall survival
NNS: Nutrition nurse specialist
ADL: Activities of daily living
